# Refining physical exercise training studies in patients with inflammatory bowel disease: patient selection and assessment of physical fitness changes

**DOI:** 10.1186/s13063-024-08163-0

**Published:** 2024-05-10

**Authors:** Karlijn Demers, Bart C. Bongers, Daisy M. A. E. Jonkers, Marieke J. Pierik, Laurents P. S. Stassen

**Affiliations:** 1 Department of Surgery, Maastricht University Medical Center+, P. Debyelaan 25, Maastricht, 6229 HX The Netherlands; 2 Department of Gastroenterology-Hepatology, Maastricht University Medical Center+, P. Debyelaan 25, Maastricht, 6229 HX The Netherlands; 3 Department of Surgery, Institute of Nutrition and Translational Research in Metabolism (NUTRIM), Maastricht University, PO Box 616, Maastricht, 6200 MD the Netherlands; 4 Department of Gastroenterology-Hepatology, Institute of Nutrition and Translational Research in Metabolism (NUTRIM), Maastricht University, PO Box 616, Maastricht, 6200 MD the Netherlands; 5 Department of Nutrition and Movement Sciences, Institute of Nutrition and Translational Research in Metabolism (NUTRIM), Maastricht University, PO Box 616, Maastricht, 6200 MD the Netherlands

With interest we read the study protocol of the IBD-FITT study by Lund et al., an open-label randomized controlled trial (RCT) investigating the effects of a 12-week aerobic moderate-intensity physical exercise training program for patients with inflammatory bowel disease (IBD) and moderate disease activity [1]. We agree with the authors regarding the potential beneficial role of physical exercise training in the holistic management of IBD. Also, robust evidence regarding its effectiveness as a standardized treatment modality for this specific population is indeed still lacking. We therefore echo the authors’ emphasis on the critical need for research on these interventions, with an RCT being a useful step in such research. However, we would like to underscore the need for accurate assessment of physical fitness components in such physical exercise training intervention studies in terms of 1) adequate patient selection, 2) monitoring the response to training, and 3) evaluating changes in physical fitness following the intervention.

Physical exercise training is, by definition, a subcategory of physical activity that is planned, structured, repetitive, and intended to improve or maintain components of health-related physical fitness, including body composition, aerobic fitness, muscular strength, muscular endurance, and flexibility [2]. Adequate patient selection based on those physical fitness components consistent with the rationale of the intervention is crucial to ensure optimal therapeutic potential of the intervention, especially considering the limited evidence for impaired health-related physical fitness in patients with IBD [3]. When investigating the effects of aerobic training, as proposed by Lund et al., inclusion of individuals with initially impaired aerobic fitness is pivotal to maximize the intervention’s potential effects. Neglecting proper patient selection may lead to inclusion of individuals with sufficient aerobic fitness, limiting the therapeutic effect and potential for improvement, which might result in an underestimation of the intervention’s true impact. The steep ramp test, a practical short maximal exercise test performed on a cycle ergometer [4], was recently found to have high criterion validity for aerobic fitness assessment in patients with IBD and can serve as an easy and valid evaluation of aerobic fitness in this population [5]. The emphasis on adequate patient selection aligns with the P4 (*Predictive*, *Preventive*, *Personalized*, and *Participatory*) health concept, reflecting a proactive, personalized, and patient-centered healthcare approach [6]. To facilitate adequate patient selection, validated screening tools (i.e., questionnaires) and easily applicable measurement methods (i.e., physical tests) for other physical fitness components (i.e., body composition, muscular strength, muscular endurance, and flexibility) are warranted in patients with IBD. Hence, future research should prioritize the development and validation of such screening tools and assessment methods.

Objective and accurate assessment of those physical fitness components targeted by the intervention throughout and after the intervention is required to gain a more profound understanding of how physical exercise training impacts physical fitness in patients with IBD. Monitoring the response to training throughout the intervention is desirable, allowing for potential adjustments to the program for individuals demonstrating limited or no response. This adaptive strategy aims to enhance the tailoring of the physical exercise training program based on individual responses, optimizing responsiveness and outcomes [7]. Lund et al.’s RCT primarily investigates the effect of exercise on disease-specific health-related quality of life [1]. However, parameters of subjective well-being, such as quality of life and fatigue, are often multifaced and are influenced by various factors. The alterations observed in subjective well-being post-intervention could be attributed to the Hawthorne effect, meaning that participants involved in physical exercise intervention studies may report more positive changes or biased responses due to increased awareness and attention, higher expectations, or a tendency to please researchers [8]. Hence, the precise mechanism underlying these changes requires a nuanced analysis. Although Lund et al. also incorporate objective secondary measurements (i.e., circumference measurements, disease activity, blood pressure, lipid status, and biomarkers), these solely provide valuable insights into the overall health effects and safety of the intervention. However, these are inadequate for evaluating the effectiveness of the physical exercise training intervention in improving aerobic fitness in patients with IBD and, in turn, improving quality of life. Within the design of such studies, we believe it is crucial to investigate whether an increase in physical fitness as a result of the intervention correlates with improved (patient-reported) health outcomes in order to provide more compelling evidence on the specific advantages of physical exercise training in the IBD population.

In developing (randomized) physical exercise training studies, we suggest to adhere to the international consensus on therapeutic exercise and training (i-CONTENT) tool to ensure high therapeutic potential of the intervention [9]. This tool, designed to assess the quality of exercise therapy programs studied in RCTs, covers critical elements such as patient selection, type and dosage of the exercise program, qualified supervision, type and timing of outcome assessment, safety, and adherence. Adhering to this tool can assist in developing high-quality exercise interventions for individuals with IBD, thereby increasing the evidence on whether exercise therapy could serve as a beneficial complement to current IBD management.

## Data Availability

Not applicable.
